# Variation of existence and location of aquaporin 3 in relation to cryoresistance of ram spermatozoa

**DOI:** 10.3389/fvets.2023.1167832

**Published:** 2023-03-28

**Authors:** Belén Pequeño, Cristina Castaño, Manuel Alvarez-Rodriguez, Paula Bóveda, María Gemma Millán de la Blanca, Adolfo Toledano-Díaz, Diego Andres Galarza, Heriberto Rodriguez-Martinez, Belén Martínez-Madrid, Julián Santiago-Moreno

**Affiliations:** ^1^Department of Animal Reproduction, National Institute for Agriculture and Food Research and Technology, Spanish National Research Council (INIA-CSIC), Madrid, Spain; ^2^Laboratorio de Biotecnología de la Reproducción Animal, Facultad de Ciencias Agropecuarias, Universidad de Cuenca, Cuenca, Ecuador; ^3^Department of Biomedical and Clinical Sciences (BKV), Obstetrics and Gynecology, Linköping University, Linköping, Sweden; ^4^Departamento de Medicina y Cirugía Animal, Facultad de Veterinaria, Universidad Complutense de Madrid (UCM), Madrid, Spain

**Keywords:** aquaporins, sperm, channels, cryoresistance, ram

## Abstract

**Introduction and objective:**

Osmotic changes during the process of freeze-thawing involve changes in the location of aquaporins (AQPs) in membrane domains of spermatozoa. Some AQPs, like aquaporin 3 (AQP3), are linked to sperm cryotolerance in the porcine species. Conspicuous individual variability exists between rams and their ejaculates, which may be classified as displaying good freezability (GFE) or poor freezability (PFE), depending on several endogenous and environmental factors. The present work aimed to examine whether differences in freezability could even involve changes in location and expression of AQP3 in ram spermatozoa.

**Methods:**

Thirty ejaculates from 10 rams (three of each) were evaluated and subsequently classified as GFE (*n* = 13) or PFE (*n* = 17) through a principal component analysis (PCA) and k-means cluster analysis. Spermatozoa were examined for the presence, abundance and distribution of AQP3 by western blot and immunocytochemistry, employing a commercial rabbit polyclonal antibody (AQP3 - ab125219).

**Results and discussion:**

Although AQP3 was found in the sperm acrosome, midpiece, principal and end piece of the tail in both fresh and after frozen-thawed samples, its highest immunolabeling was found in the mid- and principal piece. In the GFE group, the expression of AQP3 in the mid- and principal piece was greater (*P* < 0.05) in frozen-thawed samples than in fresh specimens while such differences were not detected in the PFE group. Sperm cryotolerance relates to changes in AQP3 expression and thus AQP3 could be used as a biomarker for cryotolerance.

**Conclusion:**

A greater capacity of AQP3 localization in mid- and principal piece of the spermatozoa could be linked to an increase the osmo-adaptative capacity of ejaculates with better capacity to withstand freeze-thawing processes.

## Introduction

Cryopreservation of semen is highly relevant for modern livestock breeding using artificial insemination (AI). Compared to cattle, cryopreserved semen has a limited use in sheep due to lower pregnancy rates registered after AI in contrast to the use of liquid refrigerated semen (1). Among limiting factors, there is a need for frozen-thawed semen to be deposited either deep in the cervix or intra-utero. Considering the anatomical characteristics of the ewe cervix and the difficulties to pass it (2) AI with cryopreserved ram semen is most often done by laparoscopy, a procedure not less cumbersome, restricting the wide use of this reproductive technology in farms (3). In addition, freezing procedures have not substantially improved in the last years, yielding low cryosurvival (4–6). Thus, selecting appropriate donors and ejaculates with good response to the freeze-thawing process is considered a pre-requisite in this species and a starting point for selection for freezability, a pathway as cattle has followed for decades.

Rams present high inter-individual variability for semen freezing, allowing the classification in good or poor freezability. Moreover, ejaculates from the same male have a variable cryoresistance according to several environmental (e.g., photoperiod) and endogenous (e.g., endocrine status) factors (7). Since the association between conventional sperm variables and freezability is low (8), the precise mechanisms underlying differences in cryosensitivity between individuals or ejaculates have yet to be elucidated. In this sense, recent studies highlighted the potential use of the transmembrane proteins aquaporins (AQPs), which allow the transport of water through cell membranes, as cryotolerance biomarkers in pig (9).

A group of AQPs named aquaglyceroporins—including the AQP3, AQP7, AQP9, and AQP10—play an essential role in the transport of water but also of other solutes as glycerol, urea, and other small no-electrolites, across the cellular membrane (10). Indeed, glycerol, which is the main cryoprotectant used for freezing mammal sperm, is preferably transported than water by this group of AQPs. These aquaglyceroporins are involved in the sperm response to cryopreservation procedures; a particular variation in their expression and domain location appears to be related to sperm cryoresistance in some animal species (11).

The presence and localization of AQPs in spermatozoa vary between species and cell domains. AQP3 has been identified in bull (12), boar (13), stallion (14), mouflon and ibex (15), dromedary camel (16), mouse and human (17) sperm. Prior to cryopreservation, relative amounts of AQP3 have been found to be higher in ejaculates from boar sperm with good freezability than in ejaculates with poor freezability (18). In bovine sperm, amounts of AQP3 positively correlate with motility after thawing for ejaculates with good freezability (19). In either species, AQP3 appears to be a good marker for the identification of good and bad freezers. Unfortunately, neither AQP3 nor other AQPs has been identified in ram spermatozoa (20).

Given all the above, the main objective of the present study was to define whether differences in freezability of ejaculates classified as of good (GFE) or poor freezability (PFE) could even involve changes in location and expression of AQP3 in ram spermatozoa.

## 2. Materials and methods

### 2.1. Animals, sperm collection, and cryopreservation

Ten Merino rams were housed at the Department of Animal reproduction of INIA-CSIC (Madrid-Spain, 40°25′N). They were kept in a sand-floor stable (250 m^2^) with a partial roof cover, under natural photoperiod. The rams were fed a diet that consisted of barley straw, dry alfalfa, and grain. In addition, water, vitamins, and mineral blocks were available *ad libitum*. All handling procedures were approved by the INIA Ethics Committee (reference regional government 2011/017; PROEX 154/17) and performed following the Spanish Policy for Animal Protection RD53/2013, which conforms to European Union Directive 2010/63 regarding the protection of animals used in scientific experiments.

Three ejaculates per male were collected (10:00 a.m.) at 1–3 weeks intervals, during February–March 2021 (Supplementary Tables 1, 2) with an artificial vagina using non-estrous female teasers. Therefore, a total of 30 ejaculates were collected over a period of 40 days. No ejaculates were ever discarded. Semen samples were extended in TES-Tris-glucose-based medium (TTG) containing TES (210.6 mM), Tris (95.8 mM), glucose (10.1 mM), Streptomycin (0.54 mM), Penicillin (2.14 mM), 6% egg yolk (vol/vol), and 5% glycerol (vol/vol) (pH adjusted to 6.8–7.2, osmolarity 320–345 mOsm/kg). The ejaculates were cooled to 5°C for 3 h, and loaded into 0.25 ml French straws (IMV Technologies, L'Aigle, France). Samples were kept separately for each ram, and cryopreserved following a conventional freezing in static liquid nitrogen vapor in straws (21).

All frozen sperm samples were thawed after 3 months by placing the straws in a water bath at 37°C for 30 s.

### 2.2. Sperm analysis

Samples of sperm suspensions were examined fresh and post-thawing. Sperm motility and kinetic variables were evaluated using a computer-aided sperm analysis system (CASA) (Sperm Class Analyzer^®^ v.4.0 software, Microptic S.L., Barcelona, Spain) coupled to a Nikon Eclipse model 50i phase contrast microscope with negative contrast capability, × 100 magnification. A minimum of three fields totalling 500 sperm cell tracks were examined. The following parameters were determined: total motility (%), progressive motility (%), curvilinear velocity (VCL, μm/s), straight-line velocity (VSL, μm/s), average path velocity (VAP, μm/s), the amplitude of lateral head displacement (μm) (22). Sperm viability was evaluated using fluorochrome propidium iodide (PI) (23), accounting 200 cells per sample.

Sperm cryoresistance was evaluated by calculating a cryoresistance ratio (CR) defined as CR= (post-thaw value/fresh value) × 100 (24). Retrospectively, two groups of ejaculates were classed as either of good (GFE) or poor freezability (PFE).

#### 2.2.1. Expression and localization of aquaporins

Western blot (WB) and immunocytochemistry (ICC) were used to detect the presence and distribution of AQP3 in fresh and frozen-thawed spermatozoa employing commercial rabbit polyclonal antibodies (AQP3 - ab125219 from Abcam (Netherlands) B.V). The antibody specificity was assessed using the corresponding AQP3 blocking peptide (Supplementary Figure 1). For WB analysis, proteins were extracted from 35 million spermatozoa. Seminal plasma was retrieved by centrifugation at 5,400 ×g. After two rounds of washing with phosphate-buffered saline (PBS) solution at 5,400 ×g for 5 min, the pellet was subjected to crude mechanical disruption and incubated with lysis buffer at 4°C for 60 min in agitation. The lysis buffer contains 6% sodium dodecyl sulfate (SDS), 125 mM Tris, 1 mM benzamide, 1% cocktail of the protease inhibitor, and 1 mM phenylmethylsulfonyl fluoride. The samples were centrifuged again at 5,400 *g* for 5 min, the supernatant was collected, and Laemmli sample buffer (DTT, SDS, Tris, glycerol, b-mercaptoethanol, and bromophenol blue) was added. The protein suspensions were then denatured by heating at 94°C for 4 min. Aliquots of 35 μl were subsequently loaded onto 12% SDS-PAGE gels. Electrophoresis was performed at 150 V for 90 min, then transferred the proteins to Amersham™ Protran^®^ 0.45 μm nitrocellulose membranes (Global Life Sciences Solutions, Buckinghamshire, UK) at 300 mA for 90 min. The membranes were then blocked with 5% BSA (Merck KGaA, Darmstadt, Germany) in PBS-Tween for 60 min and incubated at 4°C overnight with the primary antibodies (AQP3 - ab125219) at a dilution 1/1,000. The membranes were washed three times in PBS-Tween and incubated with the secondary antibodies (mouse anti-rabbit IgG-HRP, sc-2357) (Santa Cruz Biotechnology Inc., Dallas, TX, USA) at a dilution of 1/15,000 for 120 min at room temperature, followed by extensive washing in PBS-Tween. Finally, the membranes were revealed using WesternSure^®^ PREMIUM, LI-COR^®^ chemiluminescent substrate (Lincoln, NE, USA), employing the C-DIGIT instrument (LI-COR Bio-sciences) and analyzed by the IMAGE STUDIO 4.0 software (LI-COR Bio-sciences). Western blot of sheep liver tissue lysate (L) and mouse kidney tissue lysate (K) were performed to evaluate the specificity of the antibodies.

For ICC, freshly ejaculated and frozen-thawed spermatozoa were fixed in 4% paraformaldehyde, centrifuged (1,200 *g*, 6 min), and the pellet resuspended in PBS to prepare smears on slides. Slides were allowed to dry, washed with PBS-Tween, and blocked with 5% BSA in PBS for 60 min. After washing, the slides were incubated with the primary antibodies against AQP3 at 4°C overnight before again washing and incubating with the secondary antibody (polyclonal goat anti-rabbit Alexa Fluor 488) (Molecular Probes, Invitrogen, Carlsbad, CA, USA) diluted 1/500 in PBS containing 1% BSA, for 180 min in the dark (25). Negative controls where the sample was incubated only with the secondary antibody, omitting the primary antibody step, were included in each immunolabelling assay (Supplementary Figure 1). Controls for the specificity of AQP3 antibody were previously established in our lab, using the corresponding AQP3 blocking peptide, incubating AQP3 antibody together with AQP3 blocking peptide 5 times concentration (Supplementary Figure 1). The sperm membrane location of the AQP3 was examined by optical sectioning in fluorescence imaging (Zeiss Apotome 3) using an inverted Zeiss Axio Observer microscope at × 630 magnification, connected to a camera Zeiss Axiocam Mono. In addition, the proportion of spermatozoa showing AQP3 in different cell regions was determined (examining 200 cells) using a Nikon Eclipse E200 epifluorescence light microscope (Nikon Instruments Inc, New York, NY, USA).

### 2.3. Statistical analysis

All statistical analyses were performed using STATISTICA software for Windows v.13.3 (Tibco^®^ Inc., Tulsa, OK, USA). The values for sperm variables that showed non-normal distributions, as determined by the Shapiro–Wilk test, were arcsine-transformed before analysis. Principal component analysis (PCA) of the variance-covariance matrix for the cryoresistance ratios of the sperm variables was performed. Only those variables with a factor loading higher than 0.3 with its respective component were selected from the linear combination of variables in each component. Regression factors for each component after PCA were used for cluster analyses to identify rams as good and bad freezers and ejaculates classified as displaying good freezability (GFE) or poor freezability (PFE). The cryoresistance ratios that best explained each Principal Component were subjected to k-means cluster analysis to identify two subpopulations. STATISTICA specifically uses Lloyd's method to implement the k-Means algorithm. The right number of clusters was determined by a v-fold cross-validation algorithm in the STATISTICA package. Differences in the expression of AQP3 between groups (GFE and PFE) were compared by a *t*-test and ANOVA. Differences in the relative abundances of AQP3 bands in WB between samples with different sperm freezability were analyzed by *t*-test. Data were expressed as means ± SEM. Where applicable, significance was set at *p* < 0.05.

## 3. Results

Semen quality analysis of fresh semen samples is included in Table 1. The sperm variables that best explained each Principal Component were the cryoresistance ratio for total motility (factor loading, 0.90), for straight-line velocity (factor loading, 0.36), and for viability (factor loading, 0.83). Thus, these variables were chosen for cluster analysis and to classify the rams as good and bad freezers, and ejaculates as GFE or PFE. Cluster analysis identified seven rams as good freezers and three rams as bad freezers, but at least one ejaculate from six good freezers displayed poor freezability (Supplementary Tables 1, 2). Thus, the study focused in ejaculates rather than individuals. A total of 13 ejaculated were considered as GFE and 17 as PFE. Table 2 shows greater cryoresistance ratio values (*P* < 0.01) for viability, motility, and straight-line velocity in the GFE group than in the PFE group.

**Table 1:** Semen quality of ram freshly ejaculated spermatozoa (*n* = 30).

Total motility (%)	69.66 ± 2.43
Progressive motility (%)	46.59 ± 2.58
VCL (μm/sg)	109.82 ± 3.80
VSL (μm/sg)	4.22 ± 1.67
VAP (μm/sg)	61.60 ± 2.09
ALH (μm)	4.36 ± 0.14
Viability (%)	77.50 ± 1.22

**Table 2:** Cryoresistance ratios (mean ± SEM) for ram sperm.

**Cryoresistance ratio**	**GFE (*N* = 13)**	**PFE (*N* = 17)**
Viability	61.34 ± 5.12^a^	31.46 ± 4.78^b^
Motility	79.28 ± 9.57^a^	37.61 ± 4.05^b^
Straight-line velocity (VSL)	119.66 ± 11.84^a^	95.76 ± 9.81^b^

Ejaculates with good (GFE) or poor freezability (PFE). Superscript letters (a, b) indicate significant differences (*P* < 0.01) between groups.

**Figure 1:**
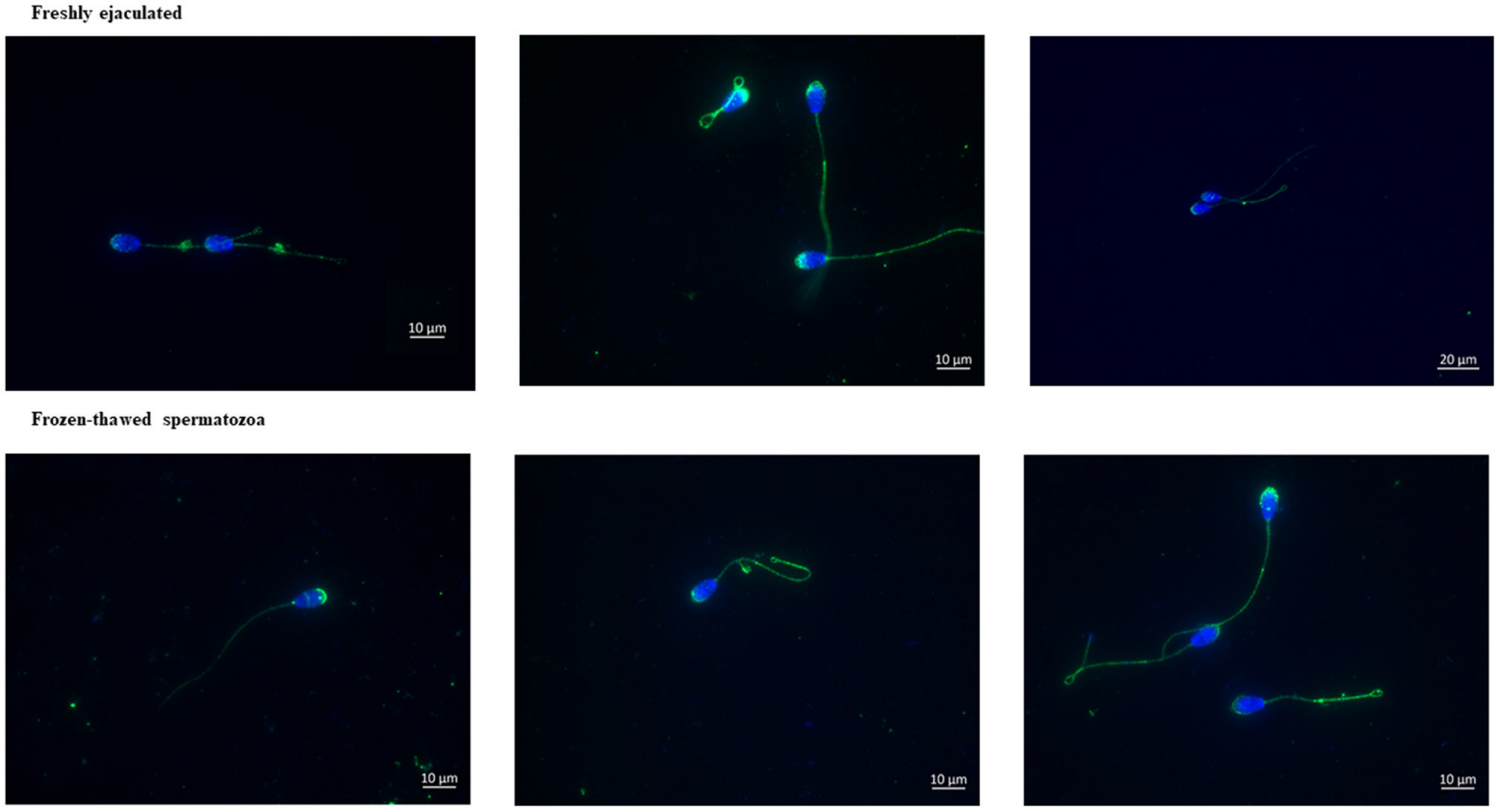
Immunolabeling of AQP3 in ram (fresh and frozen-thawed spermatozoa). AQP3 is located in the acrosome, midpiece, principal piece of the tail, and end piece of the tail.

**Figure 2:**
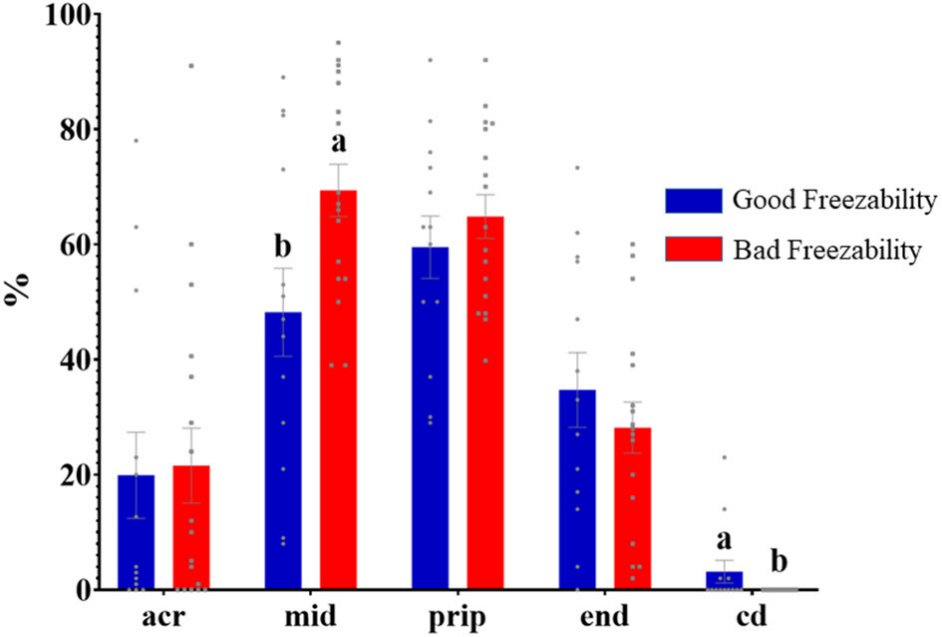
Proportion (mean ± SEM) of freshly ejaculated spermatozoa retrieved from ejaculates classified as good (GFE) or poor freezability (PFE), with representative localization patterns of AQP3 in membrane domains. Acr, Acrosome; mid, midpiece; prip, principal piece; end, end piece; cd, cytoplasmic droplet. Different letters (a, b) indicate significant differences (*P* < 0.05) between groups.

**Figure 3:**
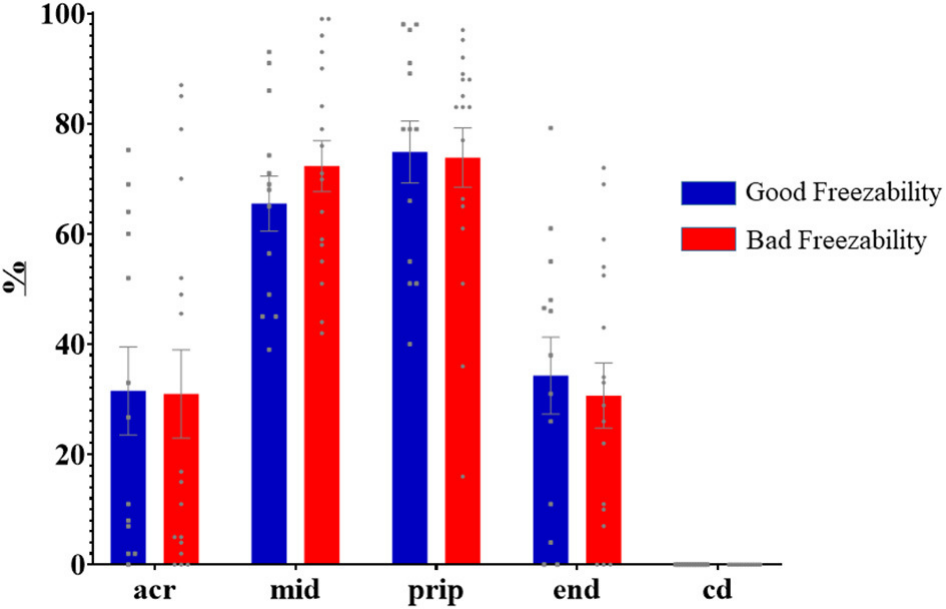
Proportion (mean ± SEM) of frozen-thawed spermatozoa from ejaculates classified as of good (GFE) or poor freezability (PFE) with representative localization patterns of AQP3 in membrane domains. Acr, Acrosome; mid, midpiece; prip, principal piece; end, end piece; cd, cytoplasmic droplet.

**Figure 4:**
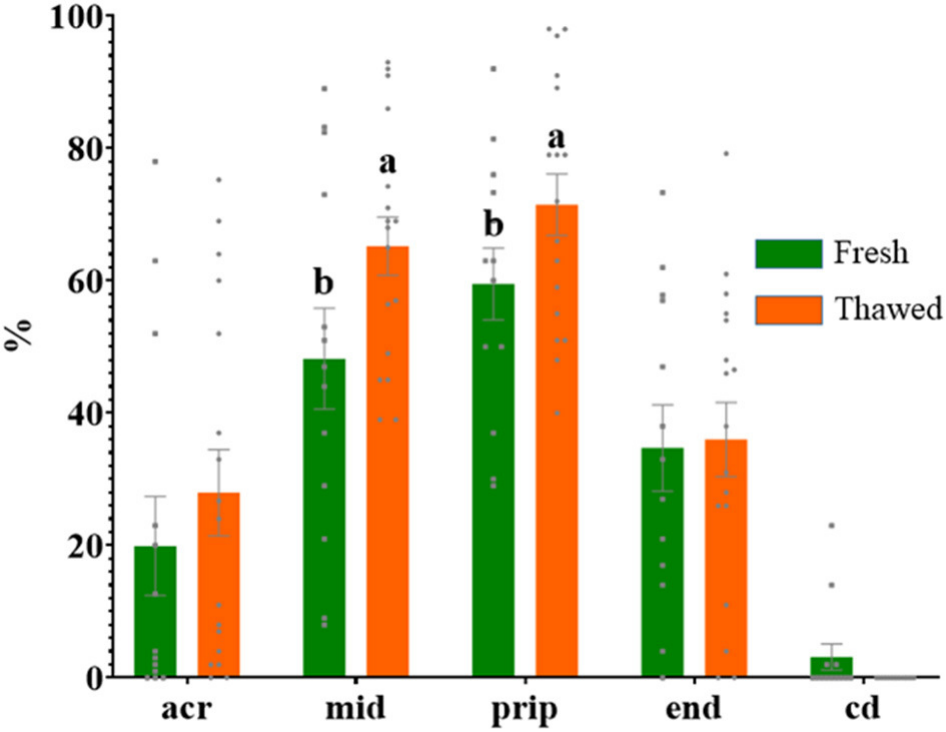
Proportion (mean ± SEM) of fresh and frozen-thawed spermatozoa from ejaculates classified as of good freezability (GFE) with representative localization patterns of AQP3 in membrane domains. Acr, Acrosome; mid, midpiece; prip, principal piece; end, end piece; cd, cytoplasmic droplet. Different letters (a, b) indicate significant differences (*P* < 0.05) between groups.

**Figure 5:**
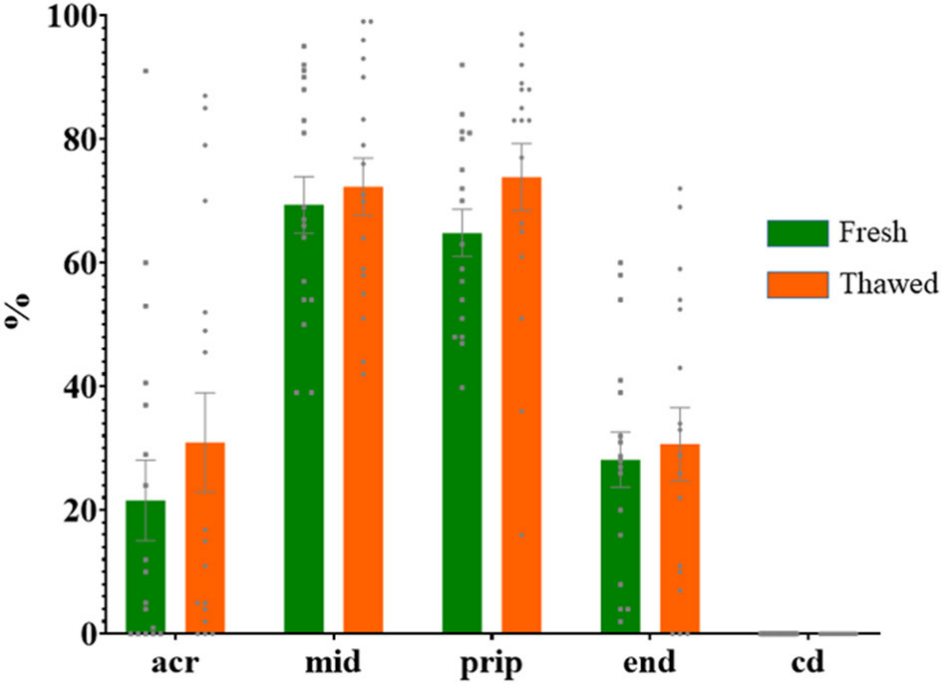
Proportion (mean ± SEM) of fresh and frozen-thawed spermatozoa from ejaculates classified as of poor freezability (PFE) with representative localization patterns of AQP3 in membrane domains. Acr, Acrosome; mid, midpiece; prip, principal piece; end, end piece; cd, cytoplasmic droplet.

Immunolabeling of AQP3 in freshly ejaculated and frozen-thawed sperm samples revealed AQP3 location in the acrosome, midpiece, principal piece, end piece, and cytoplasmic droplet (Figure 1). In fresh samples, the proportion of spermatozoa showing immunolabeling of AQP3 in the midpiece was lower in GFE than in PFE samples (Figure 2). No differences were found in frozen-thawed samples between GFE and PFE (Figure 3). The freeze-thawing process produced an increase (*P* < 0.05) of AQP3 expression in both midpiece and principal piece of GFE ejaculates (Figure 4), unlike in PFE ejaculates in which no changes were found (Figure 5).

**Figure 6:**
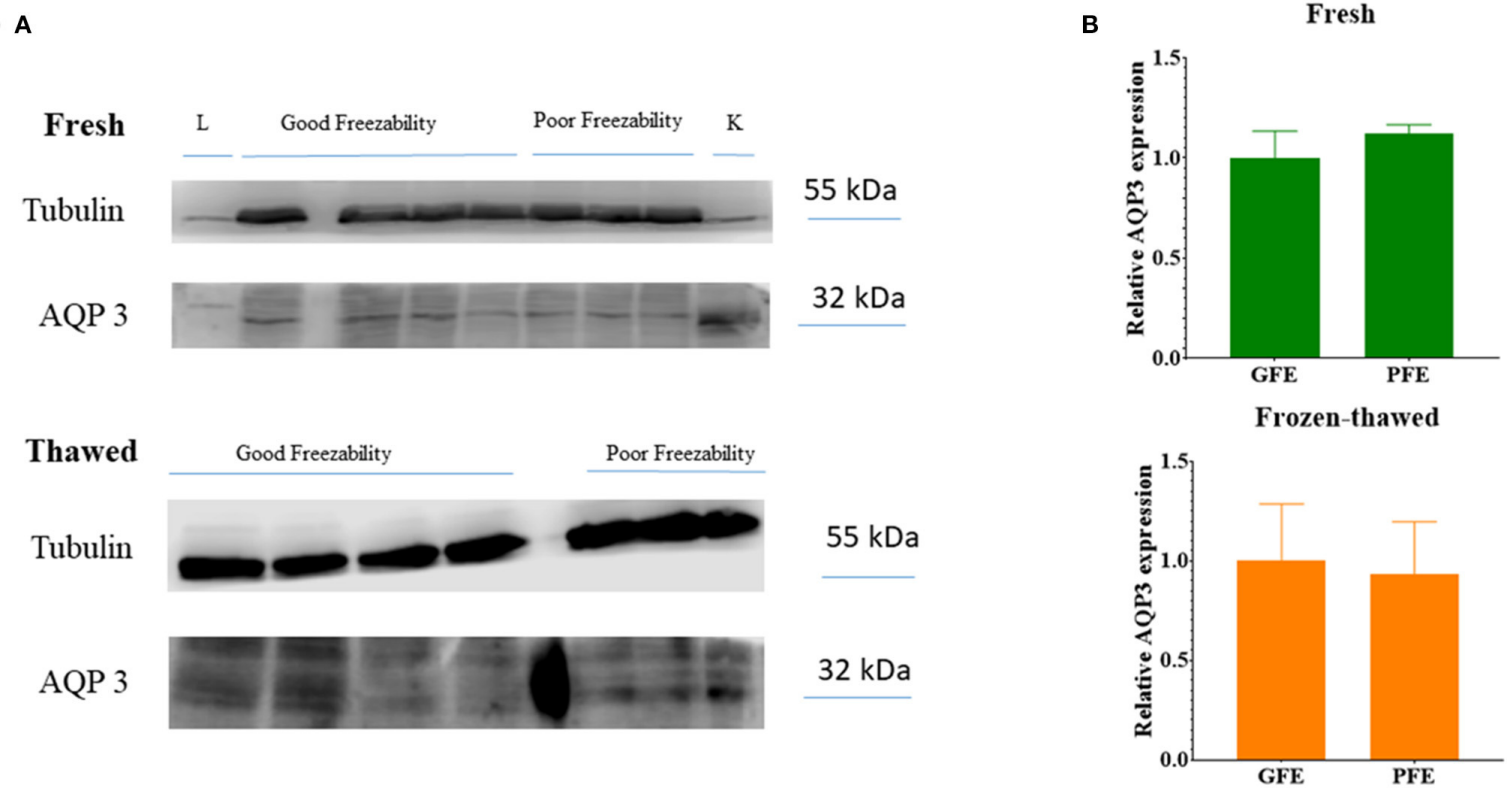
**(A)** Immunoblots for AQP3 in fresh and frozen-thawed ram spermatozoa of ejaculates classified as of good (GFE) or poor freezability (PFE). L, Liver; K, kidney. **(B)** Relative abundances of AQP3 bands (as mean ± SEM) in GFE and PFE from fresh and frozen-thawed samples. No significant differences were found between GFE and PFE after quantification of 32 kDa bands and normalization using tubulin protein as an internal standard.

WB identified the presence of AQP3 as a single band of about 32–33 kDa (Figure 6A) in either freshly ejaculated and frozen-thawed spermatozoa. Relative abundances of AQP3 bands in GFE and PFE in fresh and frozen-thawed samples did not show significant differences (Figure 6B).

## 4. Discussion

AQP3 was found in the sperm acrosome, midpiece, principal and end piece of the tail in both fresh and after frozen-thawed samples; displaying its highest immunolabeling in the mid- and principal piece. In the GFE group, the expression of AQP3 in the mid- and principal piece was greater in frozen-thawed samples than in fresh specimens while such differences were not detected in the PFE group, suggesting a relocation of AQP3.

This is the first study identifying AQP3 in ram sperm. The location of AQP3 in ram spermatozoa was similar to that seen in their wild ancestor, the mouflon (*Ovis musimon*): the acrosome, midpiece, principal piece, and end piece (15). This location has also been found in dromedary (16), and boar sperm (13). In bull, AQP3 is only detected in the midpiece (12, 19), whereas in mouse and human sperm, AQP3 is located in the principal piece (17). All these findings reveal that AQP3 location is variable, depending on the species. However, the specific role of these changes on species-specific variability of sperm cryo-resistance is yet unknown.

We found that cooling and freeze-thawing did not affect the relative abundances of AQP3, which is consistent with previous results reported in bull sperm (19). By contrast, in boar sperm ejaculates, relative amounts of AQP3 prior to cryopreservation were found to be higher in GFE than in PFE (18). These findings suggest, a priori, that the relative abundance of AQP3 is a useful cryotolerance marker in boar, unlike ram. However, many factors might affect these results; indeed, in other boar sperm study by the same research group, no differences were observed in terms of AQP3 content between GFE and PFE (13). Among the plausible factors of variation, along with differences in WB procedures (e.g., types and dilution of primary antibodies), we should consider factors that could temporarily affect AQPs location, such as fluctuations of certain hormones: thyroxine, testosterone and melatonin (15). In addition, some individuals always produce ejaculates with good or bad resistance to cryopreservation processes, but sometimes the same individual may provide ejaculates with a variable cryoresistance, according to their endocrine status, the season (7), or to unidentified factors. Curiously, in species with acceptable response to sperm cryopreservation, such as bull, there are no differences in the abundance of AQP3 between GFE and PFE (19). It is important to remember that cattle has been selected for semen freezability for nearly a century, compared to other species of livestock. In contrast, in species known as bad freezers, such as stallion and boar where such directed selection has not been done or it is incipient, these differences are manifest (14, 18). Freezability of ram sperm presents a high variability, but usually frozen-thawed sperm has low fertility rates than fresh ones (26). Thus, more studies focused on individuals and breeds with extensive sperm freezability variations in are needed to discard the abundance of AQP3 in fresh samples as a biomarker of good sperm freezability in rams.

Freeze-thawing process increased the proportion of sperm showing AQP3 in both midpiece and principal piece in GFE ejaculates, but no changes were found in PFE. By contrast, in boar sperm, no differences were seen in AQP3 distribution between GFE and PFE or between fresh and frozen–thawed samples (18). Similarly, the individual male response to the freeze-thawing process was not directly associated with the membrane localization of AQP3 in dromedary sperm (16). The mechanisms involved in how AQP3 sperm membrane pattern distribution and AQP3 relocalization after freeze-thawing process could indicate sperm cryoresistance in some species remain unclear. In wild ruminant species (ibex, mouflon), with a strong reproductive seasonality, the rutting season is accompanied by the rise of testosterone secretion which, in turn, appears to be linked with the decrease of sperm freezability and the increase of the proportion of spermatozoa with AQP3 in the midpiece (27). A greater expression of AQP3 in the midpiece of ibex and mouflon frozen-thawed spermatozoa might result in a rapid water and glycerol flux and thus major osmotic stress (15); however, this study was made exclusively with frozen-thawed samples, so it is unknown if occurred a possible relocation of AQP3. In our study, the increase of AQP3 in both midpiece and principal piece after thawing compared to fresh samples, in GFE ejaculates, might suggest a greater capacity of AQP relocation in ejaculates with better cryoresistance. These changes in membrane domain localization of AQPS might be link to the internalization of AQPs by endocytosis into intracelullar vesicles (endosomes), which transfer them to other membrane domain, or by exocytosis of dedicated storage vesicles (28). A greater capacity of AQP3 relocalization could be indicative of an increased osmo-adaptative capacity of ejaculates hence yielding a better response to freeze-thawing processes. All this might explain, at least in part, the increase of AQP3 in both mid and principal piece of GFE ejaculates. Freeze-thawing process also appears to modify domain localization of other AQPs, such as AQP7 in boar sperm, where it was equally suggested to play different roles in the osmotic regulation during the cryopreservation process (25).

WB assay showed a band of 32–33 kDa, similar than in wild small ruminants (i.e., mouflon and ibex) (15), and human, in which a dimer of 62 kDa dimer is also found (29). Similarly, in stallion sperm, two bands of 30 kDa and 60 kDa (dimer) were observed (14). Other studies showed similar results but with slight differences across mammalian species: a single band of 28 kDa in dromedary camel (16), a single band of 25 kDa in boar (18), and a band of 42 kDa in bull sperm (19). These differences reveal a species-specific influence.

In conclusion, our results confirmed changes in AQP3 area location in ram sperm as well as specific changes in AQP3 location and expression found after freeze-thawing in ejaculates considered as GFE. Such AQP3 relocalisation could be linked to an increase the osmo-adaptative capacity of ejaculates with better capacity to withstand freeze-thawing processes, suggesting AQP3 expression could be used as a biomarker for potential sperm frezability in ram semen.

## Data availability statement

The raw data supporting the conclusions of this article will be made available by the authors, without undue reservation.

## Ethics statement

The animal study was reviewed and approved by INIA Ethics Committee (reference regional government 2011/017; PROEX 154/17).

## Author contributions

BP performed the experiments, analyzed generated data, and wrote the first draft of the manuscript. MA-R, HR-M, and BM-M performed study design, data analysis, and reviewed manuscript. CC, PB, MM, AT-D, and DG contributed to the investigation and methodology. JS-M designed the experiment, data analysis, and corrected the manuscript. All authors contributed to the article and approved the submitted version.

## Supplementary material

Supplementary Figure 1 **(A)** Negative control: sample incubated only with secondary antibody, omitting the primary antibody step). **(B)** Immunofluorescence of the peptide competition assay for the anti-AQP3 antibody (AQP3 + blocking peptide). **(C)** Western blot resulting from incubations with the AQP3-blocking peptide. K: kidney mouse tissue lysate. R10, R6, R13, R17, R14, R16, R19, R8: Individual ram identification.

Supplementary Table 1 Rams classified as good or bad freezer after cluster analysis. CR, cryoresistance ratio; VSL, straight-line velocity.

Supplementary Table 2 Dates and ram identification from ejaculates classified as displaying good freezability (GFE) or poor freezability (PFE).
