# Supplementary figures and images for: Variation of existence and location of aquaporin 3 in relation to cryoresistance of ram spermatozoa
Source: Front Vet Sci. 2023 Mar 28;10:1167832. doi: 10.3389/fvets.2023.1167832 (PMC10086261; doi:10.3389/fvets.2023.1167832)

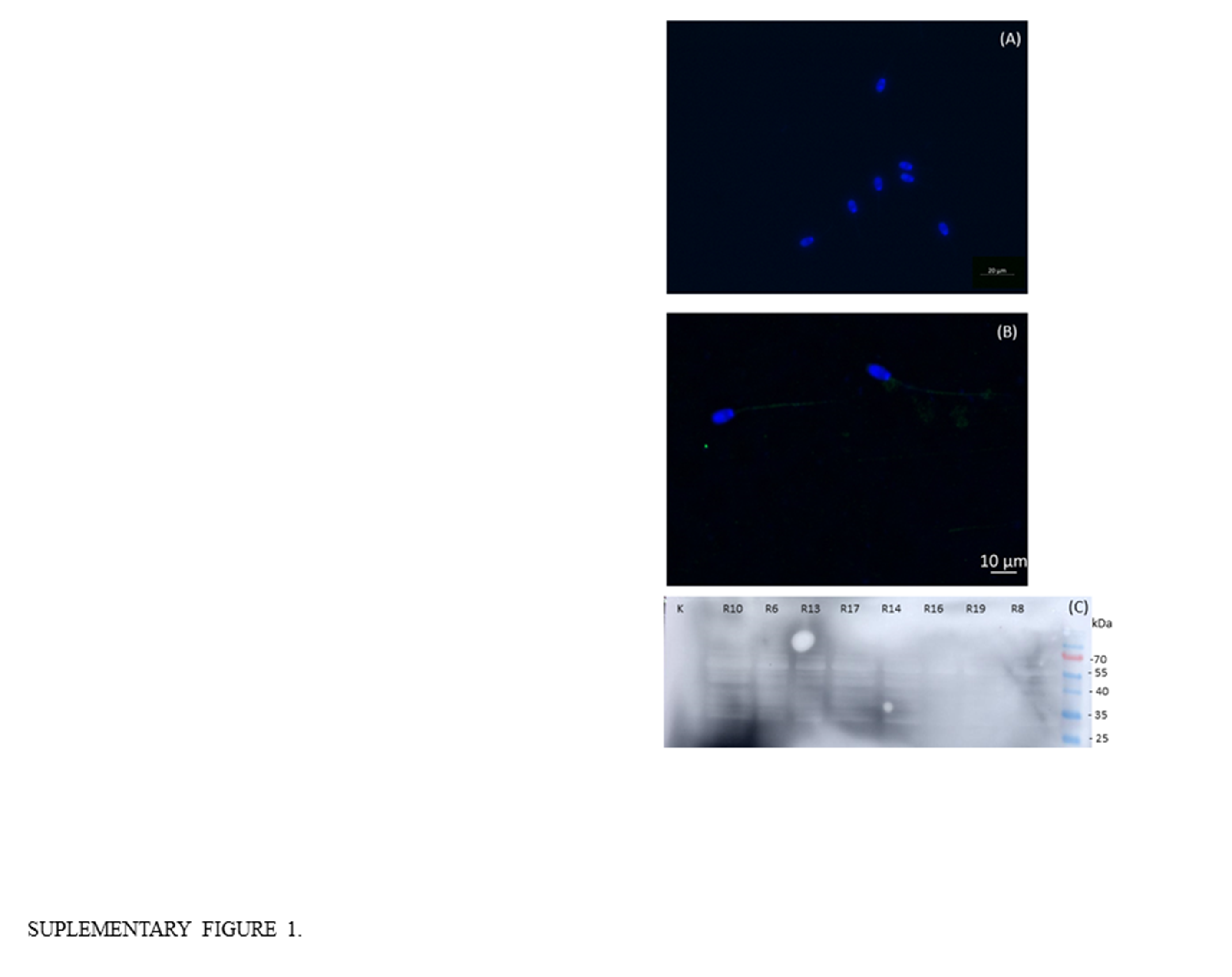

Supplement: Supplementary Figure 1 — (A) Negative control: sample incubated only with secondary antibody, omitting the primary antibody step). (B) Immunofluorescence of the peptide competition assay for the anti-AQP3 antibody (AQP3 + blocking peptide). (C) Western blot resulting from incubations with the AQP3-blocking peptide. K: kidney mouse tissue lysate. R10, R6, R13, R17, R14, R16, R19, R8: Individual ram identification. [file Image_1.TIF]
