# Supplementary material for: Variation of existence and location of aquaporin 3 in relation to cryoresistance of ram spermatozoa
Source: Front Vet Sci. 2023 Mar 28;10:1167832. doi: 10.3389/fvets.2023.1167832 (PMC10086261; doi:10.3389/fvets.2023.1167832)
Supplement: Supplementary Table 1 — Rams classified as good or bad freezer after cluster analysis. CR, cryoresistance ratio; VSL, straight-line velocity. [file Data_Sheet_1.docx]

***Supplementary Material***

**Supplementary Table 1.** Rams classified as good or bad freezer after cluster analysis. CR: cryoresistance ratio. VSL: straight-line velocity.

| **Distance** | **Good freezers** | **CR-Viability** | **CR-Motility** | **CR-VSL** | **FACTOR1** |  |
| --- | --- | --- | --- | --- | --- | --- |
| 0,412838 | Ram10 | 59,0517241 | 63,649229 | 141,311664 | 0,93655 |  |
| 0,346618 | Ram13 | 52,0661157 | 73,0074497 | 93,194635 | 0,87033 |  |
| 0,224325 | Ram14 | 60,3448276 | 48,223009 | 101,393085 | 0,29939 |  |
| 0,173788 | Ram19 | 46,1538462 | 60,2606275 | 119,186973 | 0,34993 |  |
| 0,627205 | Ram8 | 32,7433628 | 58,8993509 | 118,590176 | -0,10349 |  |
| 0,774295 | Ram80 | 39,0134529 | 52,5263097 | 86,6172925 | -0,25058 |  |
| 1,040159 | Ram9 | 75,4940711 | 72,8041955 | 91,5255438 | 1,56388 |  |
|  |  |  |  |  |  |  |
|  |  |  |  |  |  |  |
| **Distance** | **Bad freezers** | **CR-Viability** | **CR-Motility** | **CR-VSL** | **FACTOR1** |  |
| 0,266656 | Ram16 | 25,6637168 | 28,6895236 | 81,9396772 | -1,48866 |  |
| 0,020848 | Ram17 | 22,4489796 | 32,2783696 | 141,715914 | -1,24285 |  |
| 0,287504 | Ram6 | 29,3333333 | 44,0936695 | 58,7190458 | -0,93450 |  |
|  |  |  |  |  |  |  |

**Supplementary Table 2.**  Dates and ram identification from ejaculates classified as displaying good freezability (GFE) or poor freezability (PFE).

| **GFE** |  |  |
| --- | --- | --- |
| **Case** | **Animal** | **Date** |
| 1 | Ram10 | 02/03/2021 |
| 2 | Ram10 | 15/03/2021 |
| 3 | Ram10 | 30/03/2021 |
| 4 | Ram13 | 23/02/2021 |
| 5 | Ram13 | 09/03/2021 |
| 7 | Ram14 | 02/03/2021 |
| 9 | Ram14 | 30/03/2021 |
| 10 | Ram16 | 23/02/2021 |
| 16 | Ram19 | 02/03/2021 |
| 18 | Ram19 | 30/03/2021 |
| 26 | Ram80 | 09/03/2021 |
| 28 | Ram9 | 02/03/2021 |
| 29 | Ram9 | 09/03/2021 |

| **PFE** |  |  |
| --- | --- | --- |
| **Case** | **Animal** | **Date** |
| 6 | Ram13 | 23/03/2021 |
| 8 | Ram14 | 15/03/2021 |
| 11 | Ram16 | 09/03/2021 |
| 12 | Ram16 | 23/03/2021 |
| 13 | Ram17 | 02/03/2021 |
| 14 | Ram17 | 09/03/2021 |
| 15 | Ram17 | 30/03/2021 |
| 17 | Ram19 | 15/03/2021 |
| 19 | Ram6 | 23/02/2021 |
| 20 | Ram6 | 09/03/2021 |
| 21 | Ram6 | 23/03/2021 |
| 22 | Ram8 | 15/03/2021 |
| 23 | Ram8 | 23/03/2021 |
| 24 | Ram8 | 23/02/2021 |
| 25 | Ram80 | 23/02/2021 |
| 27 | Ram80 | 23/03/2021 |
| 30 | Ram9 | 30/03/2021 |
